# Surgical treatment on infective endocarditis: impact of diabetes on mortality

**DOI:** 10.1186/s12933-022-01557-x

**Published:** 2022-06-30

**Authors:** Alexander Kogan, Anat Wieder-Finesod, Jonathan Frogel, Yael Peled-Potashnik, Eilon Ram, Ehud Raanani, Leonid Sternik

**Affiliations:** 1 Department of Cardiac Surgery, Sheba Medical Center, Tel Hashomer, Affiliated to the Sackler School of Medicine, Tel Aviv University, Tel Aviv, Israel; 2 Infectious Disease Unit, Sheba Medical Center, Tel Hashomer, Affiliated to the Sackler School of Medicine, Tel Aviv University, Tel Aviv, Israel; 3 Department of Anesthesiology, Sheba Medical Center, Tel Hashomer, Affiliated to the Sackler School of Medicine, Tel Aviv University, Tel Aviv, Israel; 4 Division of Cardiology, Sheba Medical Center, Tel Hashomer, Affiliated to the Sackler School of Medicine, Tel Aviv University, Tel Aviv, Israel

**Keywords:** Infective endocarditis, Cardiac surgery, Diabetes mellitus, Epidemiology, Mortality

## Abstract

**Background:**

Type 2 diabetes mellitus (DM) is a frequent co-morbidity among patients suffering from infective endocarditis (IE). The aim of the study was to evaluate the impact of type 2 DM on the early-, intermediate- and long-term mortality of patients who underwent surgical treatment of endocarditis.

**Methods:**

We performed an observational cohort study in the large tertiary center in Israel during 14 years. All data of patients who underwent surgical treatment of endocarditis, performed between 2006 and 2020 were extracted from the departmental database. Patients were divided into two groups: Group I (non-diabetic patients), and Group II (diabetic patients).

**Results:**

The study population includes 420 patients. Group I (non-diabetic patients), comprise 326 patients, and Group II (diabetic patients), comprise 94 patients. Mean follow-up duration was 39.3 ± 28.1 months. Short-term, 30-day and in-hospital mortality, also intermediate-term mortality (1- and 3-year) was higher in the DM group compared with the non-DM group, but did not reach statistical significance: 11.7% vs. 7.7%. (*p* = 0.215); 12.8% vs. 8.3% (*p* = 0.285); 20.2% vs. 13.2% (*p* = 0.1) and 23.4% vs. 15.6% (*p* = 0.09) respectively. Long-term, 5-year mortality was significantly higher in the DM group, compared to the non-DM group: 30.9% vs. 16.6% (*p* = 0.003). Furthermore, predictors for long-term mortality included diabetes (CI 1.056–2.785, p = 0.029), as demonstrated by regression analysis.

**Conclusions:**

Diabetic patients have trend to increasing mortality at the short- and intermediate period post-surgery for IE, but this is not statistically significant. Survival of diabetic patients deteriorates after more than three years follow surgery. Diabetes is an independent predictor for long-term, 5-year mortality after surgical treatment of endocarditis, regardless of the patients age and comorbidities.

*Trial registration* Ethical Committee of Sheba Medical Centre, Israel on 02.12. 2014, Protocol 4257

## Introduction

The global burden of diabetes is rising because of increased obesity and population ageing. Infections in diabetes mellitus (DM) are relatively more common and serious. DM patients run the risk of acute metabolic decompensation during infections, and at higher risk of certain invasive infections [1]. DM is one of the most prevalent conditions in the elderly and is associated with considerable mortality, mainly from cardiovascular complications [2]. Incidence of infective endocarditis (IE), among patients with and without DM, have increased during the period 2001–2015, and significantly higher in the DM population [3]. Also, another data suggested, that prevalence of DM among patients with native valve endocarditis increased from in 22% in 2004 to 30% in 2014 [4]. In addition, recent studies have demonstrated increased mortality in diabetic patients undergoing valve surgery for IE [4–6] compared to non-diabetic patients. Surgery for IE is crucial for optimal therapy in patients with complicated endocarditis. However, the patient medical history, including DM, might influence the decision whether to operate or not. In our study, we compare the diabetic and no-diabetic patients. This information is important for risk assessment before having surgery to this population. In addition, information from various geographical areas complements our knowledge about the disease. The aim of the current study was to investigate the impact of diabetes mellitus on short and long-term mortality in a cohort of patients after surgery for IE.

## Methods

The study protocol was approved by the Sheba Medical Center Institutional Ethics Committee (Protocol No 4257). The requirement for informed consent waived because of the retrospective nature of the study. 

We carried out a retrospective, observational study of prospectively collected data from consecutive patients who underwent valve surgery for IE at a large tertiary care university hospital in Israel over a 14-year period, from 01.09.2006 and 31.08.2020. Diagnosis of IE was made according to Duke criteria [7]. Patients were divided into two groups: Group I, non-diabetic patients (non-DM), and Group II, patients with mellitus type 2 (DM). DM type 2 was defined in accordance with the American Diabetes Association as: (a) hemoglobin A1C ≥ 6.5%; (b) fasting plasma glucose levels ≥ 126 mg/d; (c) classic symptoms of hyperglycemia or a hyperglycemic crisis, a random plasma glucose level ≥ 200 mg/dL (11.1 mmol/L) [8]; or (d) currently on pharmacologic treatment (oral antihyperglycemic drugs and/or insulin). Patients under 18 years of age and patients with type 1 diabetes mellitus were excluded. Intravenous drug abuser (IVDA) patients were included in the study. Empiric antibiotic treatment was started when needed, and specific antibiotic treatment was initiated after the results of blood cultures were available, in both groups of patients, diabetic and non-diabetic. If blood cultures were negative after 72 h, specific serologic tests were done for culture negative endocarditis (*Q*-fever, *bartonella*, *Chlamydia, Brucella Legionella* and *Mycoplasma*)*.*

Using de-identified patient data from our department's database, we evaluated the following variables: gender, age, systemic and pulmonary hypertension, smoking, dialysis-dependent renal failure, New York Heart Association functional class, chronic obstructive pulmonary disease (COPD), peripheral vascular disease, cerebrovascular accident (CVA)/transient ischemic attack (TIA), left ventricular function, previous cardiac surgery, affected valve, and microorganism isolated from the blood cultures or from valve tissue. Concomitant surgery which was defined as procedure, directly not connected to endocarditis, e.g. CABG or another valve replacement or repair.

Surgical intervention was performed according to the European Society of Cardiology guidelines. Indications for surgery were as follows: first, heart failure, e.g. severe aortic regurgitation, intracardiac fistulae, pulmonary oedema and cardiogenic shock. Second, uuncontrolled infection is considered present when there is persisting infection and when there are signs of locally uncontrolled infection. The definition of persisting infection consists of fever and persisting positive cultures after 7–10 days of antibiotic treatment. Perivalvular extension, e.g. perivalvular abscess or fistula defined as locally uncontrolled infection. And third indication is prevention of systemic embolism, defined as one or more embolic events during the first 14 days of antimicrobial therapy, growing vegetation despite appropriate antimicrobial therapy or patients with vegetations > 10 mm in length [9]. After surgery, all patients were admitted to the intensive care unit (ICU) directly from the operating room. In the operating room and the ICU, patients from both groups received intravenous continuous infusion of regular insulin according to the Society of Thoracic Surgeons practice guideline [10]. After discharge from the ICU, patients were transferred either to a step-down unit or directly to the cardiac surgical department floor. Patients from Group I (non-DM) did not receive insulin or any other hypoglycemic medication. Patients from Group II (DM) were restarted on their preoperative anti-glycemic regimen (per oral drugs and/or insulin) as soon as they resumed regular eating. Antibiotic's treatment was continued at least 6 weeks from the operation.

Firstly, we evaluated the following outcome variables: 30-day, in-hospital, 1-, 3- and 5-year mortality. Secondary, we also evaluated cause of long-term mortality. CVD mortality was defined as death where CVD, e.g. ischemic heart disease (ICD codes I20–I25), cardiac failure (ICD codes I11, I13, I50), cerebrovascular disease (ICD I60–I69), peripheral artery disease (ICD I70–I79) and aortic aneurysm (ICD I71) [11]. Cancer mortality defined as death due different kind of malignancies. Pulmonary, infection-related, renal and liver causes of death defined as other.

### Database management and statistical analysis

At discharge, all patient data were reviewed, corrected and entered into a database. Descriptive statistics were used to summarize data, and numerical data were expressed as means (SD). Normality of the distribution of continuous data variables was analyzed using the Kolmogorov–Smirnov test. Since not all numerical data were distributed normally, Mann–Whitney U-tests were used to evaluate differences between groups. Differences between the frequencies of categorical variables were also estimated using Fisher’s exact test. All outcome variables were compared between whole groups of patients.

A Cox proportional hazard model was constructed to assess the association between DM and mortality. To address the differences in baseline characteristic between the groups, subjects’ propensity score is first estimated, and then the outcome is regressed on the estimated propensity score. The regression choice depends on the nature of the outcome, therefore we used logistic regression for the predicted probability, which specify the propensity score. Variables that were associated with mortality adjusted to age were included in the regression model. In addition, we included pre-specified clinically significant variables in the model. The variables included in the final model were: gender, age, DM, affected valve, previous cardiac surgery, presence of perioperative heart failure and perioperative acute kidney injury. Kaplan–Meier survival analysis was performed to compare mortality among the groups, with statistical differences tested by the log-rank test. Statistical significance was assumed when the null hypothesis could be rejected at p < 0.05. All P-values are the results of two-sided tests. Statistical analyses were conducted using R (version 3.4.1).

## Results

The study population included 420 patients. Group I (non-diabetic patients) comprising 326 patients (77.6%), and Group II (diabetic patients) comprising 94 patients (22.4%). Mean follow-up duration was 39.3 ± 28.1 months. There were no differences regarding sex, average systolic left ventricular injection fraction, NIHA functional class and incidence of native or prosthetic valve endocarditis between groups I and II. Compared with the non-diabetic group, the diabetic group patients were older, had a higher prevalence of systemic and pulmonary hypertension, peripheral vascular disease and hyperlipidemia. Two IVDA patients (0.47%) were in non-diabetic group, and not even one in the diabetic group. Diabetic patients had higher EuroSCORE values, great proportion of concomitant and annular reconstruction surgery and longer bypass and cross-clamp time (see Table 1). *Staphylococcus* species were more frequent pathogen in patients with DM, 52.1% vs. 41.4% (*p* = 0.32), however, not statistically significant. Other pathogens caused IE listed in Table 2.

**Table 1:** Patients demographic and perioperative data

	Group I (Not-DM patients)	Group II (DM patients)	*p* Value
N	326	94	
Age (years)	54.8 ± 16.1	66.4 ± 11.1	0.000
Male gender (n, %)	214 (65.6%)	61 (64.9%)	0.902
NIHA III-IV (n, %)	103 (31.6%)	37 (39.4%)	0.234
Previous cardiac surgery (n, %)	124 (38.0%)	36 (38.3%)	1.000
Native valve endocarditis (n, %)	212 (65.0%)	62 (66.0%)	0.903
EF (%)	54.6 ± 15.1	54.8 ± 15.9	0.914
Hypertension (n, %)	136 (42.0%)	76 (81.7%)	0.000
COPD (n, %)	15 (4.6%)	10 (10.8%)	0.044
Dialysis (n, %)	6 (2.1%)	5 (6.5%)	0.057
Hyperlipidemia (n, %)	100 (31.0%)	77 (81.9%)	0.000
PVD (n, %)	7 (2.2%)	12 (13.5%)	0.000
CVA/TIA (n, %)	46 (14.7%)	17 (18.5%)	0.414
Pulmonary hypertension (n, %)	40 (12.9%)	13(14.6%)	0.723
Smoking (n, %)	81 (25.3%)	35 (38.5%)	0.017
Standard EuroSCORE I	7.4 ± 3.9	10.0 ± 4.1	0.000
Logistic EuroSCORE %	16.3 ± 17.7	27.3 ± 24.4	0.000
Bypass time (min)	121.7 ± 56.1	148.8 ± 68.1	0.001
Cross-clamp (min)	86.1 ± 37.6	108.5 ± 48.3	0.000
Aortic valve involvement (n, %)	172 (52.8%)	55 (58.5%)	0.349
Mitral valve involvement (n, %)	175 (53.7%)	54 (57.4%)	0.558
Tricuspid valve involvement (n, %)	2 (0.6%)	0 (0%)	0.280
Annular reconstruction (n, %)	44 (13.5%)	21 (22.3%)	0.023
Concomitant procedure (n, %)	128 (39.3%)	54 (57.4%)	0.434

**Table 2:** Microbiologic profile

	Group I (Non-DM patients)	Group II (DM patients)	
N	326	94	
*Staphylococcus aureus* (n, %)	109 (33.4%)	42 (44.7%)	0.320
Coagulase-negative *Staphylococcus* (n, %)	26 (8%)	7 (7.4%)	
*Enterococcus* (n, %)	75 (23%)	19 (20.2%)	0.433
*Streptococcus* species (n, %)	89 (27.3%)	16 (17%)	0.063
HACEK (n, %)	3 (0.9%)	2 (2.1%)	0.454
Fungus (n, %)	1 (0.3%)	1 (1%)	0.121
*Coxiella burnetti* (n, %)	9 (2.8%)	2 (2.1%)	0.786
Gram-negative bacteria (n, %)	1 (0.3%)	1 (1%)	0.090
Negative cultures (n, %)	13 (4%)	4 (4.3%)	0.237

**Fig. 1.**
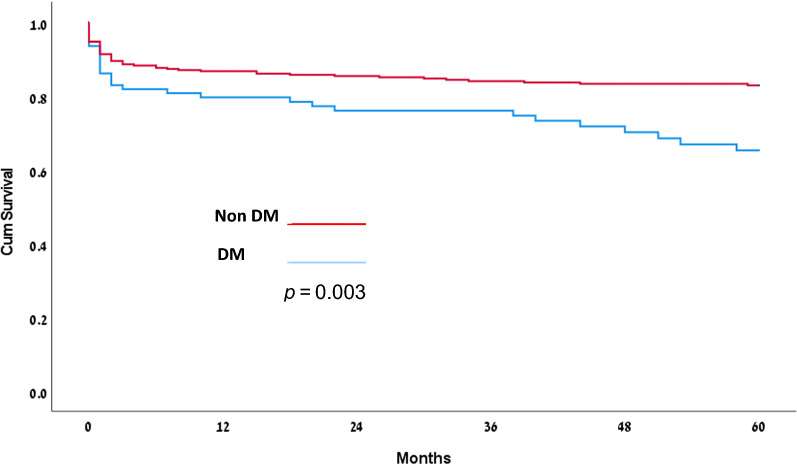
5 years Survival rate by DM groups

During study period 83 patients died, 29 (30.9%) from the DM group and 54 (16.6%) from the non-DM group. Cardiovascular mortality was significantly higher in DM group in comparison to non-DM: 65.5% vs. 38.9% (*p* = 0.017). 30-day, in-hospital, 1-year and 3-years mortality was higher in the DM group compared with the non-DM group, but did not reach statistical significance: 11.7% vs. 7.7%, (*p* = 0.12); 12.8% vs. 8.3% (*p* = 0.285); 20.2% vs. 13.2% (*p* = 0.10) and 23.4% vs. 15.6% (*p* = 0.09) respectively. Long-term, 5-years mortality was significantly higher in the DM group, compared to the non-DM group: 30.9% vs. 16.6% (*p* = 0.003) (see Table 3 and Fig. 1). By Cox analysis, we found that three factors impacted patients' 5-year mortality rates independently: diabetes mellitus (Group II), age and NIHA III-IV (See Table 4).

**Table 3:** Patient's mortality

	Group I (Non-DM) 326 patients	Group II (DM) 94 patients	*p* value
30-days mortality (n, %)	25 (7.7%)	11 (11.7%)	0.215
In-hospital mortality (n, %)	27 (8.3%)	12 (12.8%)	0.285
1-year mortality (n, %)	43 (13.2%)	19 (20.2%)	0.100
3-year mortality (n, %)	51 (15.6%)	22 (23.4%)	0.090
5-year mortality (n, %)	54 (16.6%)	29 (30.9%)	0.003
Cardiovascular mortality at 5 years (n, %)	21/54 (38.9%)	19/29 (65.5%)	0.017
Cancer mortality at 5 years (n, %)	17/54 (31.5%)	7/19 (24.1%)	
Other mortality at 5 years (n, %)	16/54 (29.6%)	3/29 (10.4%)

**Table 4:** Cox Regression for overall mortality

	B	Sig	Exp(B)	95.0% CI for Exp(B)	
				Lower	Upper
Diabetes Mellitus	0.539	0.029	1.715	1.056	2.785
NIHA III-IV	0.807	0.001	2.241	1.408	3.568
Age (Predicted probability)	− 2.213	0.013	0.109	0.019	0.632

## Discussion

Gu et al. [12] reported, that in US population, survival of diabetic subjects was lower than that of non-diabetic subjects in all age, sex, and race groups. Also, type 2 DM is associated with reduced life expectancy at almost all ages in the Scotland [13]. Adults with type 2 DM, especially those with cardiovascular comorbidity, did not live as long than their non-diabetic people in South Korea [14]. Patients with DM are at increased risk of infection. Movahed et al. [15] studied Veterans Health Administration hospitals database and reported presence of IE in 1340 (0.5%) DM patients versus 1412 (0.3%) non-DM, control group patients (relative increase of 40%), and concluded, that patients with type II DM have significantly higher prevalence of IE. Also, Abe et al. [4] identified 76,385 patients with native valve IE during 2004-14 years, from the US National Inpatient Sample, of which 22,284 (28%) had DM, and found, that proportion of DM among patients with IE increased from in 22% in 2004 to 30% in 2014 (p < 0.0001). Miguel-Yanes et al. [3] identified 16,626 patients with IE in Spain, and found, that incidence of IE, among population with and without diabetes, have increased during the period 2001–2015 with significantly higher incidence rates in the diabetic population. However, it is not clear whether this is a true increase in the incidence of IE, or a better diagnosis. In Israel, the prevalence of Type 2 diabetes was 21% among Arabs and 12% among Jews, e.g. 16.2% in the general population [16]. Prevalence of diabetic patients underwent surgery in our study 22.4%, which roughly corresponds to the prevalence of diabetes in the general population. It is interesting to note a very low frequency of IVDA, 0.47% compared to the study from New York [17], which shows a frequency of 4.3%.

Kourany et al. [18] compare outcomes between 150 diabetic and 905 non-diabetic patients with IE, from the International Collaboration on Endocarditis Merged Database. Diabetic patients underwent surgical intervention less frequently (32.0% vs. 44.9%, p = 0.003), and had higher overall in-hospital mortality (30.3% vs. 18.6%, p = 0.001). Olmos et al. [19] reported, that in-hospital mortality (43.5% vs. 30.0%; p = 0.008) were more common among patients with DM than in those without, but not described treatment modality, surgical or conservative. Farag et al. [20] studied 360 patients with IE, operated between 1993 and 2012, and reported, that non-survivors had higher rates of preoperative diabetes mellitus (*p* = 0.005), but not found diabetes as independent predictors of 30-day mortality. Yoshioka et al. [5] described 470 patients underwent valve surgery for left-sided active IE, 374 non-DM and 96 with DM. In-hospital mortality was 8% in patients without DM and 13% in patients with DM (*p* = 0.187). The overall survival rate at 1 and 5 years was 87% and 81% in patients without DM and 72% and 59% in patients with DM (*p* < 0.001). In addition, a greater proportion of patients with DM required concomitant surgery or annular reconstruction, and, as a result, a longer bypass and aortic cross-clamp time. In our patient population, we observed similar results.

Wei et al. [21] studied of 866 patients, who had been diagnosed with IE. They were divided into three groups: 469 patients in the normoglycaemia group, 246 patients in prediabetes group and 151 patients in diabetes group. Surgery was performed in 72.3%, 68.7% and 55.6% of cases respectively (*p* = 0.001). During median follow-up of 2.4 year, 126 (14.5%) died. Compared with those in the normoglycaemia group, higher long-term mortality was seen among people with prediabetes or diabetes (9.7% vs. 22.0% vs. 28.1%, p < 0.001). Lin et al. [22] described 412 patients with IE divided into 2 groups: group 1, patients with DM (n = 72) and group 2, patients without DM (n = 340) and concluded, that overall in-hospital mortality rate for both groups was 20.2% and was higher in group 1 than in group 2 (41.7% vs. 16.5%, p < 0.01). Among the entire cohort of patients in our study, the percentage of the diabetic patients was 22% and those with DM were older. These data are comparable with other studies [5, 16, 19–21]. The possible explanation for this fact is that the long-term course of diabetes is associated with a sharp increase in the frequency of IE. Østergaard et al. [23] identified 300,000 patients with DM through the Danish Prescription Registry and investigated the association between the duration of DM and the incidence of IE. In patients with DM duration of 0–5 years, 5–10 years, 10–15 years, and more than 15 years, the incidence rates of IE were 0.24, 0.33, 0.58, and 0.96 cases of IE/1000 person years, respectively. Patients with DM duration 5–10 years, 10–15 years, and > 15 years were associated with a higher risk of IE compared with DM duration 0–5 years. Another study from the above mention Danish Registry [24], included 1767 patients with IE undergoing surgery, 735 patients < 60 years (24.1% female), 766 patients 60–75 years (25.8% female), and 266 patients ≥ 75 years (36.1% female). The proportions of patients undergoing surgery were 35.3, 26.9, and 9.1% for patients < 60 years, 60–75 years, and > 75 years, respectively. Mortality at 90 days was 7.5, 13.9, and 22.3% (p < 0.001) for three age groups. In adjusted analyses, patients 60–75 years and patients ≥ 75 years were associated with a higher mortality, HR = 1.84 (95% CI: 1.48–2.29) and HR = 2.47 (95% CI: 1.88–3.24) as compared with patients < 60 years. In addition, the diabetic patients had more co-morbidities, higher operation risk and longer operation time.

Our study carried out in a contemporary cohort of Israeli patients, who underwent surgery due to IE, demonstrates several important implications regarding the impact of type 2 DM on short-, mid- and long-term mortality. Our results are very similar to previously reported: 30-day mortality 11.7% vs. 7.7%, 1- and 5-years mortality was 86.8% vs. 79.8% and 84.4% vs. 69.1% for diabetic and non-diabetic patients respectively. Diabetic patients have trend to increasing mortality at the short- and intermediate period post-surgery for IE, but this is not statistically significant. Statistically significant difference was observed only for 5-years survival (*p* = 0.003). Main cause of mortality during follow-up was cardiovascular mortality was significantly higher in DM group in comparison to non-DM: 65.5% vs. 38.9% (*p* = 0.017).

There are a few limitations in our study. First, our study is retrospective in design. Second, our study was conducted in a single-center cardiac surgery department. Third, very high-risk patients are not candidate for surgery, there might be an option, that non-operated IE patients would change the survival results. Fourth, the authors do not collect information regarding the time interval between the diagnosis and the time of the surgical intervention. All patients were operated on while performing the indications for the operation without any delay in both the diabetic and non-diabetic groups. We don’t investigate connection between early surgery and mortality.

## Conclusions

Diabetic patients have trend to increasing mortality at the short- and intermediate period post-surgery for IE, but this is not statistically significant. Survival of diabetic patients deteriorates after more than three years follow surgery. Diabetes is an independent predictor for long-term, 5-year mortality after surgical treatment of endocarditis, regardless of the patient's age and comorbidities.

## Data Availability

Data, supported the findings of this study are available from corresponding author upon request.

## Abbreviations

DM: Diabetes mellitus
CI: Confidence interval
IE: Infective endocarditis
IVDA: Intravenous drug abuser
CVD: Cardiovascular disease
COPD: Chronic obstructive pulmonary disease
CVA: Cardiovascular accident
TIA: Transient ischemic attack
CABG: Coronary artery bypass grating
ICU: Intensive Care Unit
SD: Standard deviation
NIHA: New York Heart Association
EuroSCORE: European System for Cardiac Operative Risk Evaluation
HACEK: *Haemophilus* species*, Actinobacillus actinomycetemcomitans, Cardiobacterium hominis, Eikenella corrodens, Kingella* species
